# Q fever and toxoplasmosis in South African livestock and wildlife: a retrospective study on seropositivity, sporadic abortion, and stillbirth cases in livestock caused by *Coxiella burnetii*

**DOI:** 10.1186/s12917-023-03645-w

**Published:** 2023-09-21

**Authors:** Maruping L. Mangena, Nomakorinte Gcebe, Peter N. Thompson, Abiodun A. Adesiyun

**Affiliations:** 1 Agricultural Research Council –Vaccines and Diagnostics Development Programme, Onderstepoort Veterinary Research, Private Bag X 05, Tshwane, 0110 Onderstepoort South Africa; 2 Department of Production Animal Studies, Faculty of Veterinary Science, University of Pretoria, Private Bag X04, Tshwane, 0110 Onderstepoort South Africa; 3 Agricultural Research Council – Bacteriology and Zoonotic Diseases Diagnostic Laboratory, Onderstepoort Veterinary Research, Private Bag X 05, Tshwane, 0110 Onderstepoort South Africa; 4 Centre for Veterinary Wildlife Research, Faculty of Veterinary Science, University of Pretoria, Private Bag X04, Tshwane, 0110 Onderstepoort South Africa; 5 School of Veterinary Medicine, Faculty of Medical Sciences, University of the West Indies, St. Augustine Campus, St Augustine, Trinidad and Tobago

**Keywords:** Retrospective study, Diagnostic laboratory data, Seropositivity, Risk factors, PCR detection

## Abstract

**Background:**

Q fever and toxoplasmosis are economically important zoonoses as they cause considerable losses in livestock (cattle, sheep and goats) and wildlife (antelopes, giraffes, lions, and cheetahs) through reproductive disorders such as abortions and stillbirths. Q fever and toxoplasmosis testing in South Africa is conducted by the Agricultural Research Council-Onderstepoort Veterinary Research (ARC-OVR). However, both zoonoses are understudied and not monitored in South Africa as they are not considered controlled or notifiable diseases in the Animal Disease Act 35 of 1984. A retrospective study was conducted on Q fever (2007–2009) and toxoplasmosis (2007–2017) using diagnostic laboratory data at the ARC-OVR. Also, we report on sporadic abortion and stillbirth cases in livestock from diagnostic tissue samples submitted for *Coxiella burnetii* polymerase chain reaction (PCR) detection at the ARC-OVR.

**Results:**

During 2007 to 2009, 766 animal samples were tested for *C. burnetii* antibodies and seropositivity was 0.9% (95%CI: 0.3–1.7) with sheep (1.9%; 95%CI: 0.6–4.4) having the highest seropositivity followed by cattle (0.7%; 95%CI: 0.09–2.6), while all goats (0.0%; 95%CI: 0.0–4.2) and wildlife (0.0%; 95%CI: 0.0–2.5) tested were negative. From 2007 to 2017, 567 sera were tested for *T. gondii* antibodies; overall seropositivity was 12.2% (95%CI: 9.6–15). Wildlife had highest seropositivity to *T. gondii* antibodies (13.9%; 95%CI: 9.0–19.7) followed by goats (12.9%; 95%CI: 9.2–17.4) and sheep (12.3%; 95%CI: 5.1–23.8) while seropositivity in cattle was 2.4% (95%CI: 0.06–12.9). Of 11 animals tested by *C*. *burnetii* PCR detection (2021–2022), 10 (91.0%) were positive. The amplicon sequences showed similarity to *Coxiella burnetii* strain 54T1 transposase gene partial coding sequence.

**Conclusions:**

We have confirmed the occurrence of the causative agents of Q fever and toxoplasmosis in livestock and wildlife in South Africa, with data limitations. These zoonoses remain of importance with limited information about them in South Africa. This study provides baseline information for future studies on Q fever and toxoplasmosis in South African livestock and wildlife, as well other African countries. Due to limited data collection experienced in this study, it is recommended that improvements in data collection samples tested should include associated factors such as sex, age, and breed of the animals.

## Background

Q fever is distributed worldwide except in New Zealand and is caused by *Coxiella burnetii* [1]. Q fever causes congenital effects such as late abortions, stillbirths, and endometritis in infected animals, resulting in substantial economic losses [2]. For instance, Q fever outbreaks in the Netherlands caused agricultural losses of approximately 35,000 Euro per disability-adjusted life year (DALY), indicating the economic significance of the zoonosis [3]. The most common reservoirs of *C. burnetii* are cattle, sheep, and goats, while the bacterium can also infect rodents, cats, dogs, and arthropods [4]. The most common methods for Q fever serological testing are complement fixation test (CFT), enzyme-linked immunosorbent assay (ELISA) [5], indirect haemolysis test, and immunofluorescence assay (IFA) [6]. Previously, CFT was the gold standard for Q fever diagnosis. However, lately, ELISA and IFA have replaced CFT as preferred methods for Q fever serological testing in animals due to increased sensitivity and specificity [6].

Like Q fever, prevalence of toxoplasmosis in livestock and wildlife is important because the disease is considered a public health risk in humans from consumption of raw milk and improperly cooked meat from infected animals, also causing significant economic losses [7, 8]. In Britain and Uruguay, *T. gondii* infections caused US $ 5–15 million losses annually [9]. *Toxoplasma gondii*, the causative agent of toxoplasmosis, infects a wide range of warm-blooded animals, including livestock and wildlife. Infections by the protozoan cause congenital abnormalities, late abortions and fetal death in livestock after several replication cycles of the tachyzoites [10].

Previously, toxoplasmosis diagnosis was mainly based on bioassays and microscopy, but these methods lacked sensitivity and were considered laborious and time-consuming [11]. The Sabin-Feldman test proved to be more efficient and specific. However, this test required live tachyzoites, which posed occupational hazards to laboratory workers [11]. This method was followed by the development of ELISA for serological diagnosis of toxoplasmosis. However, ELISA required species-specific antigens which might be difficult to obtain [7]. The development of direct agglutination tests, such as the latex agglutination test (LAT) and modified agglutination test [12] that used formalin-killed tachyzoites instead of live ones was the breakthrough in the veterinary diagnosis of toxoplasmosis [13]. However, lately, these techniques have become less commercially available. This led to the production of recombinant toxoplasmosis antigens in several serological assays such as LAT and ELISA, which greatly improved the diagnosis of toxoplasmosis [7, 14].

In South Africa, various laboratories, including the Agricultural Research Council-Onderstepoort Veterinary Research (ARC-OVR), are designated facilities for serological testing of both Q fever and toxoplasmosis and consequently keep records. However, currently, both Q fever and toxoplasmosis are not regularly monitored. This is because both Q fever and toxoplasmosis are not recognized as controlled or notifiable diseases in the Animal Diseases Act of 1984 (ACT 35 1984) and the Animal Diseases Regulations; R.2026 of 1986 Government in Gazette No. 10469 of 26 September 1986 [15]This is despite scientific evidence that the two zoonoses may cause huge losses through late abortions and stillbirths in livestock and wildlife [2, 7]. This means that Q fever and toxoplasmosis infections in livestock and wildlife might occur unnoticed since no routine surveillance is conducted. Furthermore, studies on Q fever and toxoplasmosis are still limited, and there are no records of retrospective studies on Q fever and toxoplasmosis in livestock and wildlife in South Africa. Thus, the study aimed to determine the occurrence of *C. burnetii* (2007–2009) and *T. gondii* antibodies (2007–2017) in various provinces of South Africa by analyzing and reporting on Q fever and toxoplasmosis serological data in the ARC-OVR database. The study also reported on factors associated with seropositivity, such as the origin of samples, species and year of testing. We also analyzed diagnostic tissue samples submitted for *C. burnetii* PCR detection for possible sporadic abortion and stillbirth cases in livestock caused by *C. burnetii* infections.

## Results

### Demographic distribution of samples

**Fig. 1:**
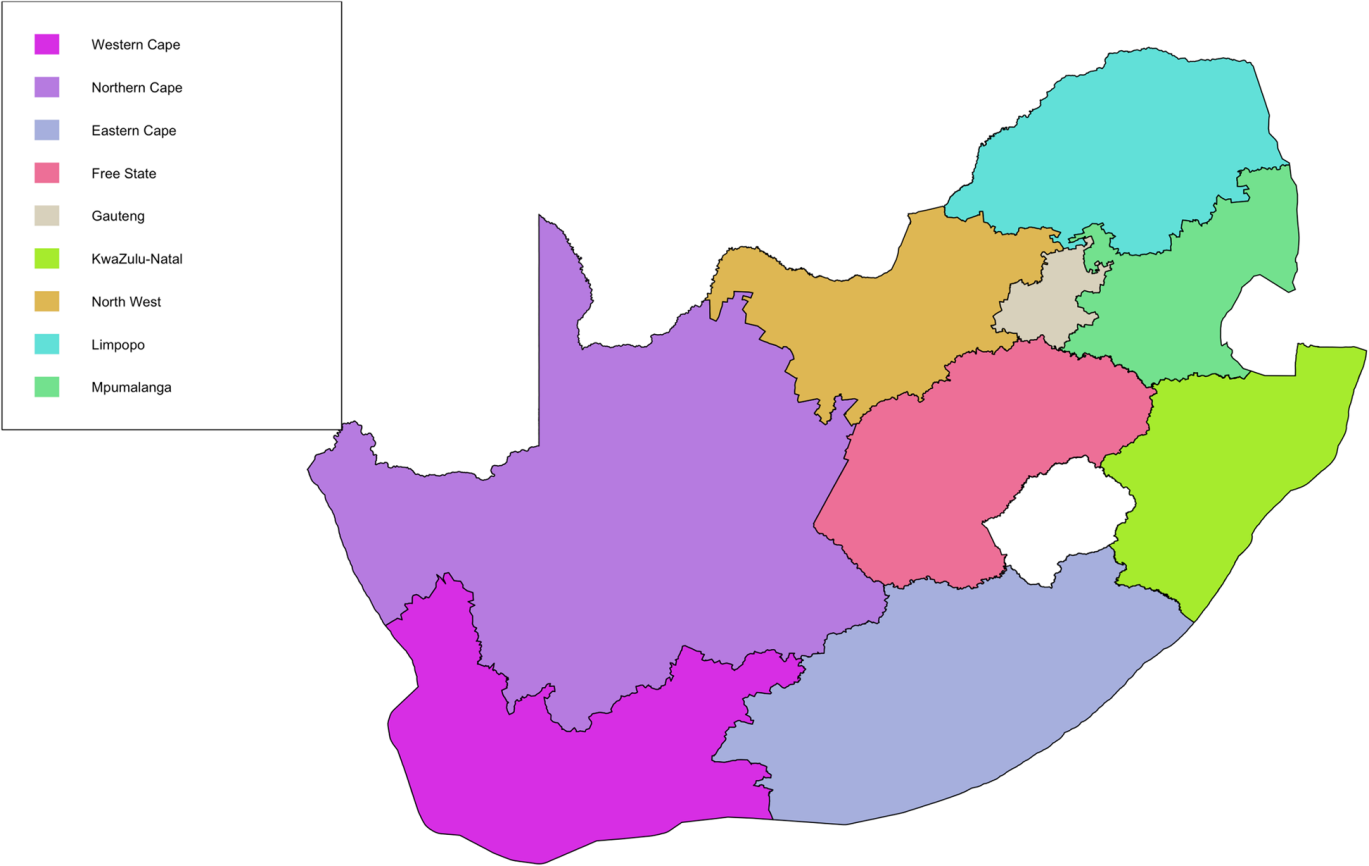
Map of South African provinces showing origins of sera tested for Q fever (2007–2009) and toxoplasmosis (2007- 2017) serology testing

For seropositivity to *C. burnetii* antibodies, between 2007 and 2009, 766 sera were tested using CFT (Fig. 1). Most of the sera submitted for Q fever testing were from cattle (35.5%), closely followed by sheep (34.3%), while the fewest samples were from goats (11.2%) as shown in Table 2. A large proportion of sera (42.7%) were tested for seropositivity to *C. burnetii* antibodies in 2008, with the least tested in 2009 (22.7%) and 2007 (21.6%), as demonstrated in Table 2. For toxoplasmosis, a large proportion of the sera tested was from goats (49.4%,) followed by wildlife (30.9%) while 2.4% of the sera were from other species (Table 3 and Fig. 1).

The most frequent origin of diagnostic tissue samples (n=11 animals) for testing by *C. burnetii* PCR was the Eastern Cape (4/11), followed by Gauteng and North West Provinces (2/11) as shown in Table 1. Small ruminants (sheep and goats) accounted for most of animals tested for *C. burnetii* positivity by PCR (8/11) while cattle accounted for the rest. Most of the animals tested were due to abortions (9/11) while 2/11 were because of stillbirths (Table 1).

**Table 1:** Tissue samples submitted for *C. burnetii* PCR detection at ARC-OVR between March 2021 and April 2022

Sample number	Number of samples (n)	Sample type	Species	Province	Reason (s) for testing
2271(a)	1	Aborted foetus (pooled liver, spleen, lungs)	Caprine	Mpumalanga	Abortion
2271 (b)	1	Aborted foetus (pooled liver, spleen, lungs)	Caprine	Mpumalanga	Abortion
5030	1	Aborted foetus (pooled liver, spleen, lungs)	Caprine	Eastern Cape	Abortion
5269	1	Pooled aborted foetus and placenta	Caprine	Eastern Cape	Abortion
12,072(a)	1	Aborted foetus (pooled liver, spleen, lungs)	Caprine	Eastern Cape	Abortion
12,072(b)	1	Aborted foetus (pooled liver, spleen, lungs)	Caprine	Eastern Cape	Abortion
12,904(a)	1	Liver from stillborn calf	Bovine	Free State	Stillbirth
12,904(b)	1	Lung from stillborn calf	Bovine	Free State	Stillbirth
12,904(c)	1	Kidney from stillborn calf	Bovine	Free State	Stillbirth
12,904(d)	1	Spleen from stillborn calf	Bovine	Free State	Stillbirth
4243	1	Aborted foetus (pooled liver, spleen, lungs)	Ovine	Gauteng province	Abortion
4322(a)	1	Liver from aborted foetus	Ovine	KwaZulu-Natal	Abortion
4322(b)	1	Lung from aborted foetus	Ovine	Kwazulu-Natal	Abortion
4322(c)	1	Pooled lung and liver from aborted foetus	Ovine	Kwazulu Natal	Abortion
4460(a)	1	Aborted foetus (pooled liver, spleen, lungs)	Caprine	Eastern Cape	Abortion
4460(b)	1	Aborted foetus (pooled liver, spleen, lungs)	Caprine	Eastern Cape	Abortion
6450	1	Placenta from aborted foetus	Bovine	North West province	Abortion
6451(a)	1	Spleen from aborted foetus	Ovine	Gauteng province	Stillbirth/ Suspected outbreak
6451(b)	1	Liver from aborted foetus	Ovine	Gauteng province	Stillbirth/Suspected outbreak
2208	1	Aborted foetus (pooled liver, spleen, lungs)	Bovine	North West province	Abortion

### Seropositivity to *C. burnetii* and *T. gondii* antibodies and risk factor analysis

There were no significant differences in seropositivity to *C. burnetii* antibodies among species (*p* = 0.22) and provinces (*p* = 0.39), while the differences were substantial between years of testing (*p* = 0.001), being highest in 2007 (Table 2). There was no association between seropositivity to *T. gondii* antibodies and years of testing (*p* = 0.13), while there were significant differences between provinces (*p* < 0.001), with the highest odds of seropositivity in Limpopo, Western Cape and Free State (Table 3). Compared to cattle, which showed the lowest seroprevalence the likelihood of seropositivity to *T. gondii* antibodies was significantly higher in goats (*p* = 0.01) and sheep (*p* = 0.04).

**Table 2:** Univariate analysis of Q fever DLD and associated risk factors for seropositivity at ARC-OVR Bacterial Serology laboratory from 2007–2009

Variable	Level	Number of samples (n)	Prevalence (%)	95%CI^a^	*p*-value
**Species**					0.22
	Bovine	272	0.7	0.09–2.6	
	Caprine	86	0.0	0.0–4.2	
	Ovine	263	1.9	0.6–4.4	
	Wildlife	145	0.0	0.0–2.5	
**Province**					0.39
	Eastern Cape	101	1.0	0.03–5.4	
	Free State	194	0.0	0.0–2.0	
	Gauteng	57	0.0	0.0–6.3	
	KwaZulu-Natal	173	1.2	0.1–4.1	
	Limpopo	63	1.6	0.04–8.5	
	Mpumalanga	34	0.0	0.0–10.3	
	Northern Cape	23	0.0	0.0–14.8	
	Western Cape	121	2.5	0.5–7.1	
**Year (s)**					0.001
	2007	165	3.0	1.0–6.9	
	2008	427	0.0	0.0–0.9	
	2009	174	0.8	0.1–2.9	
					0.3–1.7
**Total**		766	100	0.9	

CI^a^ Confidence interval

**Table 3:** Univariate and multivariable analysis of toxoplasmosis DLD and associated risk factors for seropositivity at ARC-OVR EPV laboratory from 2007–2017

Variable	Level	Number of samples (n)	Prevalence (%)	95%CI^a^	*p-*value	Odds ratio	95%CI^a^	*p-*value
**Species**					0.13			
	Bovine	41	2.4	0.06–12.9		1(base)		
	Caprine	280	12.9	9.2–17.4		41.5	2.5699.8	0.01
	Ovine	57	12.3	5.1–23.7		11.8	1.1126.6	0.04
	Wildlife	175	13.7	9.0–19.7		1.3	0.07–24.7	0.86
	Other^b^	14	7.1	0.18–33.9		3.7	0.18–76.0	0.84
**Province /Area**					< 0.001			
	Eastern Cape	23	0.0	0.0–14.8		^c^		
	Free State	141	16.3	10.6–23.5		534.7	16.3–17,515.9	< 0.001
	Gauteng	122	1.6	0.2–5.8		1(base)		
	KwaZulu-Natal	15	6.7	0.17–31.9		81.9	2.7–2480.7	< 0.001
	Limpopo	158	20.3	14.3–27.4		15.9	3.5–71.5	< 0.001
	Mpumalanga	28	7.1	0.9–23.6		20.0	1.8–226.1	0.02
	North West Province	21	0.0	0.0–16.1		^c^		
	Northern Cape	12	0.0	0.0–26.5		^c^		
	Western Cape	47	19.1	9.1–33.3		161.5	11.1–2341.5	< 0.001
**Period**					0.13			
	2007–2010	222	14.4	10.1–19.7		1(base)		
	2011–2014	202	11.9	7.8–17.8		1.4	0.4–4.9	0.62
	2015–201	143	9.1	4.9–15.0		1.8	0.5–7.1	0.39
**Total**		567	12.2	9.6–15.1				

CI^a^ Confidence interval, ^b^other (Porcine (10), Canine (2), Feline (1), Equine (1), ^c^Omitted due to perfect prediction of outcome

### PCR detection of *C. burnetii*

**Fig. 2:**
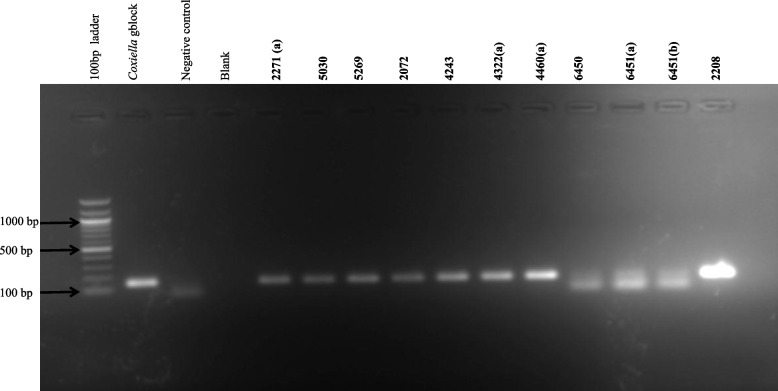
Detection of *C. burnetii* in diagnostic tissue sample by IS*1111* PCR. The first lane is Quick-Load® 100 bp DNA Ladder (New England Biolabs, Massachusetts, USA). The *Coxiella* gene fragment (gblock) from Integrated DNA Technologies (IDT, Iowa, USA) was used as template positive control while distilled water used as template negative control in the reaction. The blank is an empty unloaded lane. Samples 2271(a), 2271(b), 5269, 5030, 12,072(a), 12,072(b), 4234, 4322(a), 4322(b), 4322(c), 4322(d), 4460(a), 4460(b), 6450, 6451(a) and 6451(b)are diagnostic tissue samples submitted for *C. burnetii* PCR detection at Bacterial PCR laboratory, ARC-OVR

Detection of *C. burnetii* by IS*1111* PCR showed that samples from 10/11 animals were positive (Fig. 2). Sequence analysis of the IS*1111* PCR products revealed sequence similarity with *C. burnetii* strain 54T1 transposase gene partial coding sequence (MT268532.1) (Table 4).

**Table 4:** *Coxiella* PCR detection results and sequence confirmation of the diagnostic tissues

Sample ID	PCR result	Sequence identity	Percentage identity	E-value	Accession length (bp)	Genbank accession number
2271(a)	Positive	*C. burnetii* strain 54T1 transposase gene, partial cds^a^	93.1%	6e-34	547	MT268532.1
2271(b)	Positive	*C. burnetii* strain 54T1 transposase gene, partial cds	93.1%	6e-34	547	MT268532.1
5030	Positive	*C. burnetii* strain 54T1 transposase gene, partial cds	93.9%	1e-35	547	MT268532.1
5269	Positive	*C. burnetii* strain 54T1 transposase gene, partial cds	98.9%	1e-40	547	MT268532.1
12,072(a)	Positive	*C. burnetii* strain 54T1 transposase gene, partial cds	98.1%	1e-40	547	MT268532.1
12,072(b)	Positive	*C. burnetii* strain 54T1 transposase gene, partial cds	98.1%	1e-40	547	MT268532.1
12,904(a)	Negative	^b^	^b^	^b^	^b^	^b^
12,904(b)	Negative	^b^	^b^	^b^	^b^	^b^
12,904(c)	Negative	^b^	^b^	^b^	^b^	^b^
12,904(d)	Negative	^b^	^b^	^b^	^b^	^b^
4243	Positive	*C. burnetii* strain 54T1 transposase gene, partial cds	94.7%	6e-34	547	MT268532.1
4322(a)	Positive	*C. burnetii* strain 54T1 transposase gene, partial cds	96.8%	1e-35	547	MT268532.1
4322 (b)	Positive	*C. burnetii* strain 54T1 transposase gene, partial cds	96.8%	**-**1e-35	547	MT268532.1
4322 (c)	Positive	*C. burnetii* strain 54T1 transposase gene, partial cds	96.8%	**-**1e-35	547	MT268532.1
4460 (a)	Positive	*C. burnetii* strain 54T1 transposase gene, partial cds	91.5%	-1e-32	547	MT268532.1
4460 (b)	Positive	*C. burnetii* strain 54T1 transposase gene, partial cds	91.5%	1e-32	547	MT268532.1
6450	Positive	*C. burnetii* strain 54T1 transposase gene, partial cds	93.4%	7e-32	547	MT268532.1
6451(a)	Positive	*C. burnetii* strain 54T1 transposase gene, partial cds	93.6%	1e-32	547	MT268532.1
6451(b)	Positive	*C. burnetii* strain 54T1 transposase gene, partial cds	93.6%	1e-32	547	MT268532.1
2208	Positive	*C. burnetii* strain 54T1 transposase gene, partial cds	97.1%	3e-40	566	MT462981.1

^a^coding DNA sequence

^b^No result

### Discussion and conclusions

In this study, we obtained and analyzed data from ARC-OVR laboratories to establish the occurrence of the causal agents of both Q fever and toxoplasmosis in South Africa. Currently, in South Africa, both zoonoses are not regarded as notifiable or controlled diseases; therefore, there is no continuous surveillance for these zoonoses [15]. However, in other countries such as the USA, Q fever testing is a requirement for the export and import of livestock such as cattle and sheep [16], while it is not the case in South African livestock and wildlife. This is the first retrospective report on Q fever in South Africa. Seroprevalence of Q fever was reported by [17] to be 7.8% in cattle in the then Transvaal, now Gauteng province, while [18] and [17] reported Q fever seroprevalences in Mpumalanga and Gauteng provinces respectively, providing evidence of *C. burnetii* infections in South Africa. Despite this evidence, information on Q fever remains limited, considering that only ARC-OVR in South Africa was conducting serological testing which was stopped in 2009. Moreover, the few samples submitted for Q fever serology in this study between 2007 and 2009 indicate that the disease is not considered significant in South Africa. Also, the limited or lack of information about the reasons for submitting samples for Q fever testing further shows that there is limited knowledge on Q fever in South African livestock. Furthermore, there is scientific evidence that Q fever causes significant losses in livestock and wildlife resulting in substantial economic losses [18, 19]. Q fever was first reported in humans in South Africa in 1950 [20]. Lately, [21, 22] it is recommended that the occurrence of Q fever be continuously monitored and the relevant data accurately recorded in the testing laboratories to better understand the status of the disease in the country. The provision of an accurate database on Q fever will facilitate the decision-making process on the potential continuous surveillance of the zoonosis in South Africa.

Like Q fever, toxoplasmosis is not continuously monitored in South Africa despite evidence that the disease is present in the country. There are few reports on toxoplasmosis in the country, dating back to 2007 when [23] reported a seroprevalence of 5.6% in sheep and 37.0% in cats in 2015 [24]. Recently, [25] reported a seroprevalence of 32.6% in cattle sampled in Mpumalanga province. Moreover, between 2007 and 2017, only 567 animal samples were submitted to ARC-OVR for testing, further showing that the zoonosis is not considered significant in South Africa as only serological testing is conducted. However because toxoplasmosis is not listed as a notifiable or controlled disease in South Africa, there is no continuous surveillance. Also limited knowledge about the disease to due limited studies on toxoplasmosis might be the cause of the low flow of samples. Thus this study seeks to create awareness about the existence and toxoplasmosis in South Africa and consequences of infections. There is also evidence that toxoplasmosis infections may cause congenital disorders such as abortions in infected animals, resulting in significant economic losses in livestock and wildlife sector [26]. On the basis of the current results only, we cannot recommend that toxoplasmosis be included in the notifiable and controlled disease register in South Africa. More studies are required. However, continuous surveillance and record-keeping is required across different laboratories in South Africa so that disease is routinely monitored.

In the study, we detected C*. burnetii* in 10/11 animals tested by IS*1111* PCR. The high detection frequency in the present study may be because samples submitted are from animals that displayed possible clinical symptoms of *C. burnetii* infection, such as abortions and stillbirths [2] and may not reflect the true PCR prevalence of the disease. However, the PCR data confirms the presence of C*. burnetii* in various parts of South Africa, particularly in North West, Kwazulu-Natal, and Eastern Cape provinces where this is the first report on the zoonosis in South African livestock and wildlife. Another study by [27] also reported PCR *C. burnetii* frequency of detection from aborted materials from livestock in Iran, suggesting that *C. burnetii* PCR detection may be multifaceted and affected by risk factors such as location or origin of specimen. Other studies have reported similar findings elsewhere. For instance, [28] reported *C. burnetii* PCR detection in aborted goat material and cattle. Moreover, [27] also reported a *C. burnetii* detection (100.0%) in goat abortion material, cattle and sheep (21.3%), in Iran, which is consistent with observations in the current study of the samples that tested positive by *Coxiella* IS*1111* PCR. Most tissues tested in the study were from goats and sheep as compared to cattle and mainly due to abortion cases as compared to cattle which were stillbirth cases. This finding may suggest that *C. burnetii* infections may be responsible for abortion cases in small ruminants such as goats and sheep as compared to cattle as previously observed by [17] in the Free State province. Moreover, South Africa still has many undiagnosed abortion and stillbirth cases, caused possibly by C*. burnetii* infections. This is because state authorities usually focus on controlled diseases like brucellosis and chlamydiosis in cases of abortions, until recently where samples from some abortion cases are also submitted for *C. burnetii* PCR detection with positive results. This may reflect that although Q fever is not yet considered a notifiable or controlled disease in South Africa, there is progress in the knowledge of the disease. However, more studies need to be conducted. Although this was a national study from all nine provinces of South Africa, it is based on unrelated past and current data and the total number of animals is limited, which is one of the limitations of the study.

The study demonstrated the presence of the causative agents of both Q fever and toxoplasmosis in South Africa, laying a foundation for more studies on these zoonoses. Q fever and toxoplasmosis are important and should be regularly monitored. Proper record-keeping in various laboratories should be practised, and the records should be readily accessible. Diagnostic tissue samples were submitted for *C. burnetii* PCR detection because animals were showing clinical symptoms such as abortions or stillbirths. Thus, *C. burnetii* PCR detection frequencies in the current study do not reflect the true prevalence of the disease in the country; however, it confirms the existence of *C. burnetii* infections specifically in areas where this is the first report on the bacterium. This will pave the way for future in-depth epidemiological studies.

## Materials and methods

### Study design

The study design was to collect and analyze diagnostic laboratory data (DLD) from the ARC-OVR database of samples tested for seropositivity to *C. burnetii* and *T. gondii* antibodies and also to investigate sporadic abortion and stillbirth cases in livestock caused by *C. burnetii* using tissue samples submitted for *C. burnetii* PCR detection. Q fever and toxoplasmosis DLD were obtained from the ARC-OVR Bacterial Serology and Epidemiology, Parasites, and Vectors (EPV) laboratories, respectively. Animal samples tested included cattle, sheep, goats, and dogs. Antelope, giraffe, lion, and cheetah serum samples were grouped and referred to as wildlife. Due to low numbers, pigs (10), dogs (2), cats (1), and horses (1) were grouped together and collectively referred to as other species. Diagnostic tissue samples submitted for *C. burnetii* PCR detection were obtained from the Bacterial PCR laboratory at the ARC-OVR and analyzed for their places of origin, species, and tissues submitted as well as the reason(s) for testing (Table 1).

### Study area

South Africa is situated on the southern tip of Africa and has an area of 1,221,037 km^2^. The country has nine provinces with approximately 59 million human population (Fig. 1). The ARC-OVR Bacterial Serology and EPV diagnostic laboratories are situated in Gauteng, the smallest province in South Africa.

### Sampling

The samples were from farms, veterinary clinics, and provincial veterinary laboratories. The samples were submitted for testing for various reasons, including diagnostic, breeding, and screening, and to meet mandatory export requirements. The retrieved data consisted of tests conducted on sera, results of tests, origin of samples, year of sampling, and species. Other risk factors such as the age of animals, sex, and reasons for testing could not be obtained as the information was not included in the databases and the sample submission forms. Information on the exact origins of the samples in the form of postal codes or geographic coordinates was not available.

For PCR detection of *C. burnetii*, 20 diagnostic tissue samples from 11 animals submitted for *C. burnetii* PCR detection were obtained from the Bacterial PCR laboratory, ARC-OVR (2021–2022). These samples were composed of tissues from different species and provinces of South Africa and submitted for various reasons such as stillbirth and abortion cases, as described in Table 1.

### Laboratory serology test data

Complement fixation test (CFT), which has relative sensitivity (Se) of 99.96% in cattle and specificity (Sp) of 99.94% relative to ELISA in ruminants, was used to test for *C. burnetii* antibodies. This method also has relative Se of 26.56% and Sp of 99.97% relative to ELISA in cattle [29]. Card agglutination test (CAT) which has relative sensitivity (Se) of 100% and specificity (Sp) of 94.3% was used to test for *T. gondii* Antibodies [30]. This technique was employed using the BIO-RAD PASTOREX™ TOXO 100 antibody test kit (BIO-RAD, California, USA) according to the manufacturer ‘s instructions. Positive and negative controls were supplied with the test kit. Briefly, a drop of positive control, negative control sera, and 15 µL of the sera to be examined were applied to different fields of the agglutination card without touching each other. A diluent drop was then applied to each area on the card, followed by the addition of the latex solution. The cards were then agitated for 5 min, and the results read. The formation of a green background with red aggregates indicated that the serum contained *T. gondii* antibodies while a brown homogenous suspension showed a negative result [31].

### Statistical analysis

The data were collected and filtered using Microsoft Excel version 2016 and analyzed using Stata 15 (StataCorp, College Station, diagnostic laboratory data, USA). We assessed univariate associations of species, province of origin and year of sampling with Q fever and toxoplasmosis seropositivity using a two-tailed Fisher’s exact test. The same three variables were then included in multiple logistic regression models to adjust for confounding; however, multivariable analysis was not possible for Q fever seropositivity due to extensive collinearity. Model fit was assessed using the Hosmer–Lemeshow goodness-of-fit test.

### PCR detection of *C. burnetii*

#### DNA extraction and PCR for detection of *C. burnetii*

PCR confirmation was conducted for all diagnostic tissue samples from 11 animals. The diagnostic tissue samples consisted of various tissue specimens from different species originating from other provinces of South Africa, as shown in Table 1. Tissue samples were cut into small pieces and 10 g from each sample in placed 10 mL ice-cold buffered phosphate saline (PBS) pH 7.4 in 50 mL bead ruptor homogenizing tubes containing 2.8 mm ceramic beads. The tissue samples were then homogenized using the automated BEAD RUPTOR ELITE Bead Mill homogenizer (Omni International, Georgia, USA). The tissues were homogenized at a speed of 3 m/s for 90 s. DNA extraction from the homogenates was conducted using the Qiagen DNeasy® blood and tissue kit as previously described [17]. The homogenates were centrifuged for 15 min at 4000 rpm, and 200 µL of the supernatant was transferred to 2 mL centrifuge tubes. To the tubes, 180 µL of tissue lysis (ATL) buffer and 20 µL of proteinase K were added, suspension vortexed, and incubated at 56 °C overnight. After overnight incubation, 200 µL of lysis (AL) buffer was added, and the suspension vortexed for 15 sand incubated at 70 °C for 10 min. Absolute ethanol (200 µL) was added to the mixture, vortexed, and transferred to DNeasy® spin columns. The columns were washed twice with wash buffers; AW1 and AW2, respectively. DNA was eluted from the columns with 200 µl of elution buffer (AE).

PCR for detection of *C. burnetii* in tissues (liver, spleen, kidney, placenta) was conducted in a 50 µL reaction targeting the multi-copy transposase gene in insertion element; IS*1111* using primers 5’CGCAGCACGTCAAACCG3’ and 5’TATCTTTAACAGCGCTTGAACGTC3’ [4, 30]. The *Coxiella* gene fragment (gblock) from Integrated DNA Technologies (IDT, Iowa, USA) was used as template positive control while distilled water used as template negative control in the reaction. The reaction mixture contained 400 nM of each primer (IS*1111*F and IS*1111*R), 25 µL of the Amplicon 2 × Taq DNA polymerase Master Mix Red (Amplicon A/S, Odense, Denmark), and 10 µL of the extracted DNA. PCR amplification was conducted using BIO-RAD T100™ thermal cycler (BIO-RAD, California, USA). Cycling conditions consisted of initial denaturation at 95 °C for 15 min, 35 cycles of denaturation at 95 °C for 30 s, annealing at 60 °C for 30 s, and extension at 72 °C for 60 s for 35 cycles. The final extension was carried out at 72 °C for 10 min, and amplicons were visualized on a 1.5% *w/v* ethidium bromide-stained agarose gel with an expected size of 146 bp [32] estimated using Quick-Load® 100 bp DNA Ladder (New England Biolabs, Massachusetts, USA).

#### Sequence confirmation of *C. burnetii*

PCR confirmation of 16 tissues from 10/11 animals; 2271(a), 2271(b), 5030, 5269, 12,072(a), 12,072(b), 4243, 4322(a), 4322(b), 4322(c), 4460(a), 4460(b), 6450, 6451(a), 6451(b) and 2208 was conducted using Sanger sequencing. The IS*1111* PCR products of the 16 tissues were sent to Inqaba Biotechnical Industries (Pty) Ltd (Pretoria, South Africa) for sequencing by Sanger. Both reverse and forward PCR primers were also used as sequencing primers Sequences were manually edited using the BioEdit Sequence alignment editor (version 7.2.5) and analyzed using the BLAST search online tool (http://www.ncbi.nlm.nih.gov/blast**).**

### Study limitations

1. Factors such as the age of animals, sex, and breed could not be determined as this information was also missing from the databases and sample submission forms.
2. We did not investigate subclinical infections of *C. burnetii* and histopathological changes. This is because clients only submitted tissues for PCR detection of *C. burnetii*, and only pathogen DNA and not the disease was detected. However we confirmed the bacterium by sequencing. Some of the tissues are the ones that tested negative for other abortifacient pathogens such as brucellosis and Chlamydiosis.
3. We did not rule out co-infections because we did not investigate or confirm the cause of abortions, only focusing on *C. burnetii* detection and confirmation by sequencing.
4. Information about the reasons for submission of the animal samples for testing was not available in the database, and sample submission forms resulted in difficulties in establishing whether sampling was random or biased. This is because animal samples can be submitted for testing for various purposes such as symptoms, export, import, diagnostic, and screening, resulting in sample bias which would not be considered representative samples. Therefore, the results do not reflect true seropositivity to *C. burnetii* and *T. gondii* antibodies.

## Data Availability

In order to protect the privacy and confidentiality of clients who submitted the sera and tissues for Q fever and toxoplasmosis testing, has been de-identified.

## Abbreviations

ARC-OVR: Onderstepoort Veterinary Research
PCR: Polymerase chain reaction
IS*1111*: Insertion element of the transposase gene
DALY: Disability-adjusted life year
*C. burnetii*: *Coxiella burnetii*
CFT: Complement fixation test
ELISA: Enzyme-linked immunosorbent assay
IFA: Immunofluorescence assay
T. gondii: Toxoplasma gondii
US $: United States Dollar
LAT: Latex agglutination test
CAT: Card agglutination test
PBS: phosphate- buffered saline
DLD: Diagnostic laboratory data
EPV: Epidemiology, Parasites, and Vectors
USA: United States of America
TX: Texas
DNA: Deoxyribonucleic acid
DALRRD: Department of Agriculture, Land Reform and Rural Development
AEC: Animal Use Ethics Committee
SA: South Africa
